# NTDs in the Heart of Darkness: The Democratic Republic of Congo's Unknown Burden of Neglected Tropical Diseases

**DOI:** 10.1371/journal.pntd.0002118

**Published:** 2013-07-25

**Authors:** Anne W. Rimoin, Peter J. Hotez

**Affiliations:** 1 Department of Epidemiology, University of California, Los Angeles School of Public Health, Los Angeles, California, United States of America; 2 National School of Tropical Medicine, Department of Pediatrics and Molecular Virology & Microbiology, Baylor College of Medicine, Houston, Texas, United States of America; 3 Sabin Vaccine Institute and Texas Children's Hospital Center for Vaccine Development, Houston, Texas, United States of America; 4 James A. Baker III Institute for Public Policy, Rice University, Houston, Texas, United States of America

*The Democratic Republic of Congo may have one of the world's highest burdens of neglected tropical diseases, but nationwide there is a dearth of surveillance activities and available epidemiologic data about these conditions.*

**Figure 1:**
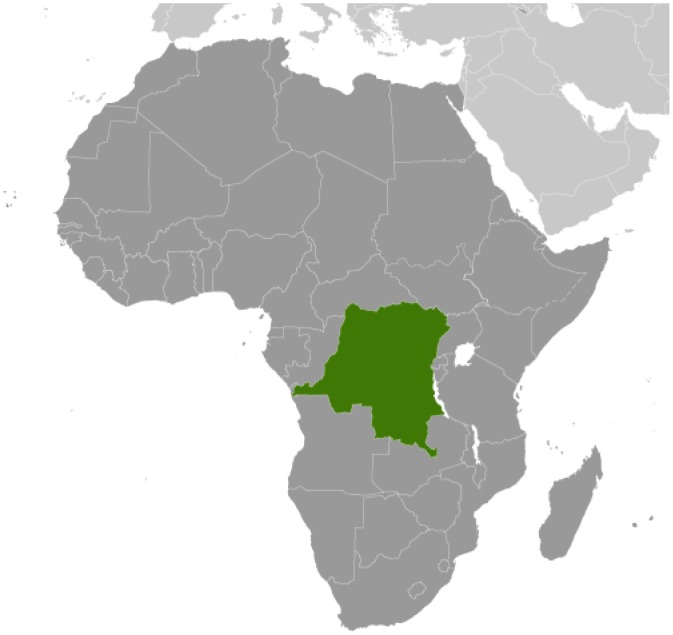
Map and location of the Democratic Republic of Congo. From CIA Factbook, https://www.cia.gov/library/publications/the-world-factbook/maps/cg_largelocator_template.html, accessed December 31, 2012.

The Democratic Republic of Congo (DR Congo) is a vast nation of almost one million square miles (about the size of Greenland or one-quarter the size of the United States, and the second largest country in Africa) and more than 70 million people located in the heart of Africa (Figure 1). For at least the last hundred years, the neglected tropical diseases (NTDs) have made a significant impact on the history of the Congo Basin. Despite a navigable river system, early European attempts to explore the Belgian Congo during the nineteenth and early twentieth centuries were blocked because of the high prevalence of human African trypanosomiasis (HAT) and other NTDs [1]. Indeed, the horrific effects of epidemic sleeping sickness were described in detail by Joseph Conrad in *Heart of Darkness* [2].

When it was known as Zaire during the last quarter of the twentieth century, the resulting dissolution of health systems and public health infrastructure contributed to the reemergence of HAT [3]. In 1998, the year following the overthrow of Mobutu Sese Seko, the number of reported cases peaked at 26,000 (the highest in the world) [3]. Even today, the DR Congo has the greatest number of cases of HAT anywhere in Africa and worldwide [4], [5], as the nation has yet to recover from years of conflict, extreme violence, and massive human migrations [6].

Beyond sleeping sickness, the decades of conflict and breakdowns in health infrastructure have contributed to the high prevalence of several major NTDs [6], although paradoxically conflict may not be a significant factor in the emergence of malaria and HIV/AIDS in DR Congo [7], [8]. In Table 1 we attempted to summarize the limited information currently available for the DR Congo. Our best information to date suggests that DR Congo may have the largest number of leprosy cases in Africa, and possibly the second or third highest number of cases of several high-prevalence NTDs, including intestinal helminth infections, lymphatic filariasis, and schistosomiasis [9], [10]. Because of its huge jungle population of nonhuman primates and other potential and actual animal reservoir hosts, the DR Congo is also believed to be the origin of several important emerging viruses of humans. According to some investigators, human HIV/AIDS first emerged in DR Congo [11], [12], as did strains of Chikungunya virus [13], Crimean-Congo hemorrhagic fever (CCHF, [14]), Ebola virus [15], monkeypox [16], and a novel rhabdovirus (Bas-Congo virus) [17].

**Table 1:** NTDs in DR Congo^1^.

Disease	Estimated Number of Cases	Rank in Africa
Hookworm Infection	31 million^2^	2^nd^
Schistosomiasis	15 million^3^	3^rd^
Ascariasis	23 million^2^	3^rd^
Trichuriasis	26 million^2^	2^nd^
Lymphatic Filariasis	49 million at risk^4^	2^nd^
Human African Trypanosomiasis	10,269–18,592^5^	1^st^
Leprosy	3,621^6^	1^st^

1 Modified from [10].

2 de Silva NR, Brooker S, Hotez PJ, Montresor A, Engles D, et al. (2003) Soil-transmitted helminth infections: updating the global picture. Trends Parasitol 19 (12): 547–551.

3 Steinmann P, Keiser J, Bos R, Tanner M, Utzinger J (2006) Schistosomiasis and water resources development: systematic review, meta-analysis, and estimates of people at risk. Lancet Infect Dis 6(7): 411–425.

4 World Health Organization (2011) Available: http://www.who.int/neglected_diseases/preventive_chemotherapy/lf/db/index.html?units=minimal&region=all&country=cod&countries=cod&year=2011. Accessed 20 June 2013.

5 Higher number from [5].

6 World Health Organization (2012) Global leprosy situation 2012. Wkly Epidemiol Rec 87: 317–328.

**Figure 2:**
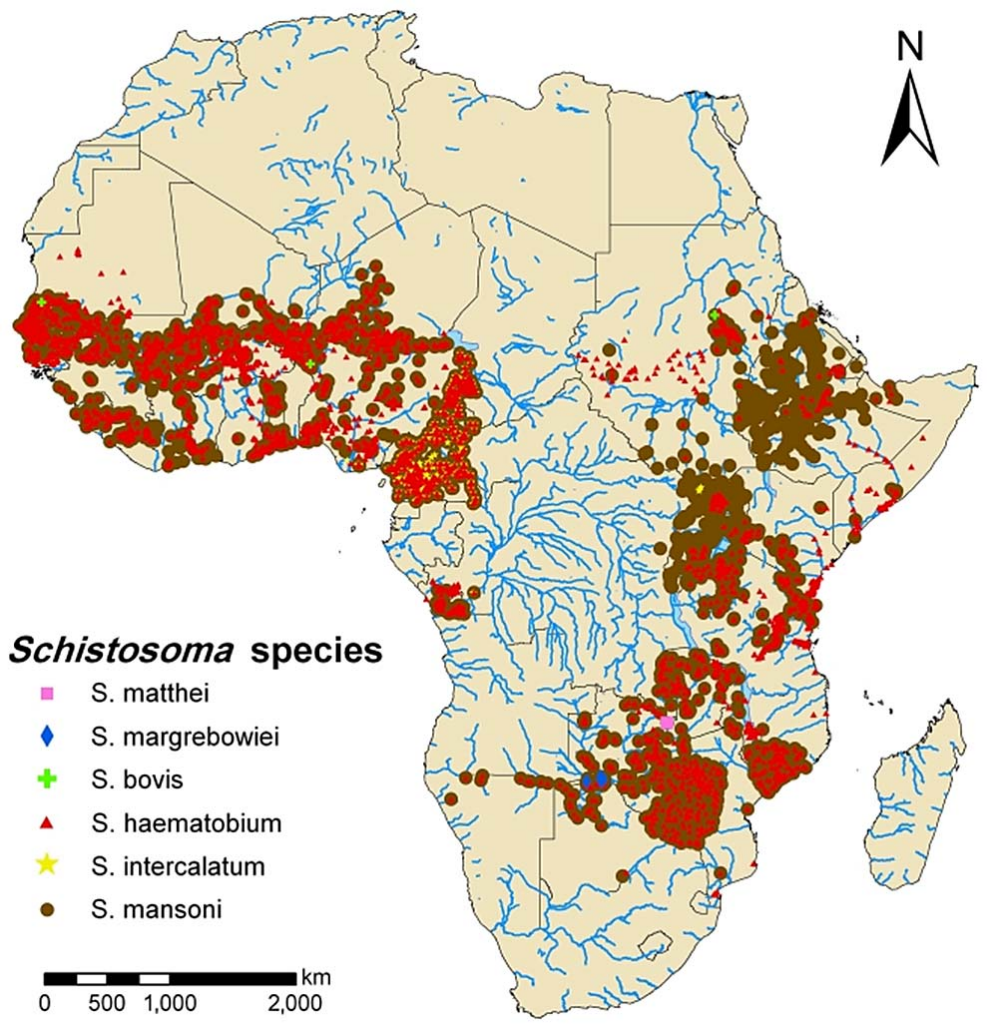
Distribution of African schistosomiasis survey locations published by Hürlimann *et al.* in *PLOS Neglected Tropical Diseases* in 2011 [18] **.**

**Figure 3:**
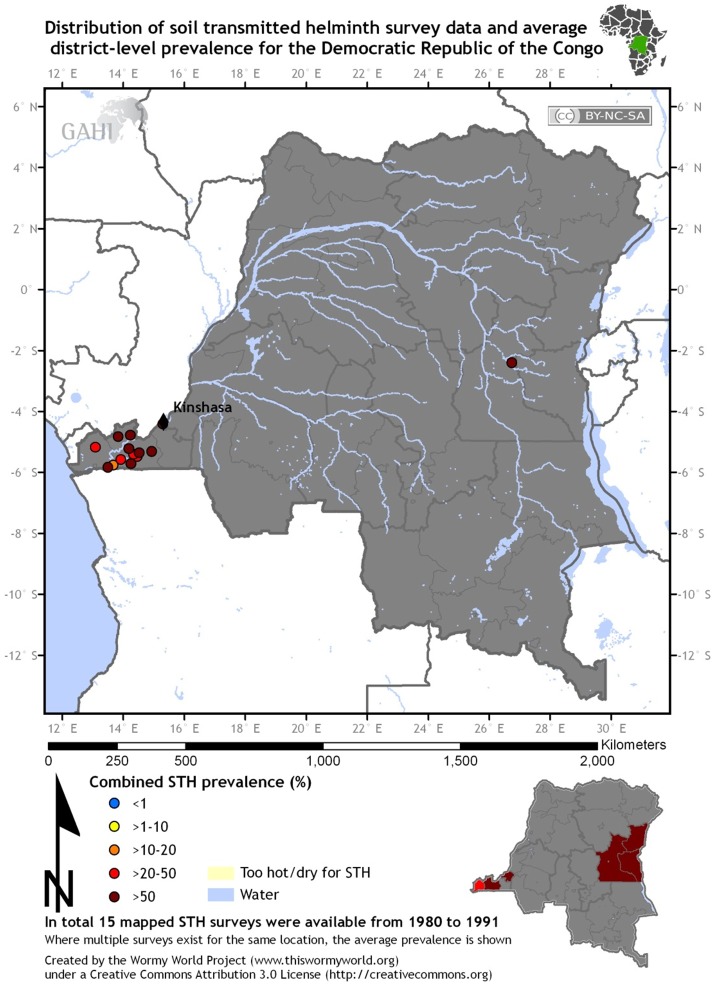
Distribution of soil-transmitted helminth infections in DR Congo. Map from Global Atlas of Helminth Infections, http://www.thiswormyworld.org/maps/democratic-republic-of-the-congo, accessed December 29, 2011.

Despite the public health importance of DR Congo's NTDs, there is much more that we do not know than we do know about both the high-prevalence NTDs and the emerging viruses in the Congo Basin. Shown in Figure 2 is a recent map of the distribution of African schistosomiasis survey locations published by Hürlimann *et al.* in *PLOS Neglected Tropical Diseases* in 2011 [18]. More than 90% of the world's cases of schistosomiasis are currently found in sub-Saharan Africa. What is striking is the dearth of schistosomiasis surveillance activity in Central Africa and particularly in the DR Congo. A similar situation is also apparent for the soil-transmitted helminth infections. Shown in Figure 3 is a map from the Global Helminth Atlas of the distribution of ascariasis, trichuriasis, and hookworm in DR Congo [19]. The map reveals a reasonable level of sampling at the mouth of the Congo River and near the capital, Kinshasa, but only a single surveillance site in the interior of the country—a country as vast as Greenland! Therefore most of our estimates for schistosomiasis and soil-transmitted helminthiases disease burdens in DR Congo are based on an extreme paucity of surveillance data, particularly active surveillance targeted to high-prevalence areas. Much the same is true for ongoing polio surveillance efforts in the country [6].

The absence of NTD disease surveillance is itself a threat to the people of the DR Congo and indeed to global public health. Time and again DR Congo has shown us how this nation represents the cradle of a number of important emerging and reemerging viral infections [11]–[17], including some with pandemic potential. Epidemic infections there have impeded polio elimination efforts just as they previously thwarted vaccine eradication campaigns for smallpox more than 30 years ago [6]. The high prevalence rate of NTDs, together with emerging viral infections, suggests that DR Congo is also a key sentinel site to detect the emergence of important coinfections, such as HIV/AIDS and schistosomiasis [20].

There is an urgent need to implement a comprehensive program for disease active and targeted surveillance comprised of studies to examine the high-prevalence NTDs, including the major helminth infections (e.g., intestinal helminth infections, schistosomiasis, lymphatic filariasis, and onchocerciasis) and key protozoan and bacterial NTDs (e.g., intestinal protozoan infections and toxoplasmosis, leprosy, Buruli ulcer, and typhoidal and non-typhoidal salmonellosis) [10]. While the Ministry of Public Health for the DR Congo deserves important credit for its key efforts to fight NTDs such as HAT, lymphatic filariasis, ochocerciasis, and schistosomiasis [21], they could be expanded and better supported by international donors. Equally important are studies to monitor the emergence of key viral infections, including arboviral infections (e.g., yellow fever, dengue, Chikungunya, and CCHF), filoviruses, poxviruses, rhabdoviruses, and retroviruses. Geographic information systems and remote sensing will become important tools for this expanded program targeting both the high-prevalence NTDs and emerging viruses [22], [23].

Studies by a number of investigators have demonstrated the feasibility of conducting high-level and sophisticated disease surveillance in DR Congo. While working there is challenging, as it is in many conflict and post-conflict sub-Saharan African countries, the Ministry of Public Health of DR Congo has time and time again demonstrated initiative and willingness to expand NTD disease surveillance and control activities. Indeed, through support of the United States Agency for International Development (USAID), DR Congo is poised to begin an ambitious program of NTD mapping and integrated disease control focused on mass drug administration for a number of its highest prevalence diseases [24]. Implementation of an ambitious NTD control program in DR Congo portends exciting possibilities to reduce the prevalence and intensity of many of the high-prevalence NTDs there, thus making a huge impact on the burden of disease in sub-Saharan Africa. The World Health Organization and its regional office for Africa are also actively working with the DR Congo government to improve health through control of NTDs and other infections [25], while the Belgian Development Agency is actively committed to rural development [26]. In parallel, there are equally compelling reasons to expand disease surveillance for the emerging viruses in DR Congo, which represent imminent threats to the populations of Africa and may ultimately exhibit pandemic potential [27]. Expanded programs for health systems research strengthening and operational research are also urgently needed and would make an important contribution toward addressing the Millennium Development Goals.
